# Comparative floral spur anatomy and nectar secretion in four representatives of Ranunculaceae

**DOI:** 10.1007/s00709-015-0794-5

**Published:** 2015-03-15

**Authors:** Sebastian Antoń, Magdalena Kamińska

**Affiliations:** Department of Botany, University of Life Sciences in Lublin, Akademicka 15, 20-950 Lublin, Poland

**Keywords:** Cell ultrastructure, Cuticle micro-channels, Holocrine secretion, Nectary structure, Morphology and anatomy, Secretory structures

## Abstract

Nectaries are common in Ranunculaceae. These secretory structures, however, have not been studied in detail despite their importance in plant-animal interactions, and data relating to the structure of nectary spurs, which are so characteristic of several genera of this family, remain scarce. In order to redress this imbalance, we sought, in the present paper, to analyze the anatomical and ultrastructural organization of the nectary spurs of four representatives of Ranunculaceae, i.e., *Aconitum lycoctonum* L., *Aquilegia vulgaris* L., *Consolida regalis* Gray, and *Delphinium elatum* L. Nectary spurs were examined using light, fluorescence, scanning electron, and transmission electron microscopy. The floral nectaries of *A. lycoctonum* and *A. vulgaris* are situated at the apices of the spurs, whereas in *C. regalis* and *D. elatum,* the nectary is located along the floor surface of the spurs. Nectar in *C. regalis* and *D. elatum* is exuded through micro-channels in the cuticle, whereas in *A. lycoctonum* and *A. vulgaris*, it is released by means of cell wall disruption, indicating that the method of nectar secretion here is holocrine. Structurally, the nectary of all four investigated species is quite similar, and its cells are typical of nectar-producing cells described in the literature. It is proposed that in *A. lycoctonum* and *A. vulgaris*, disruption of the cell wall and the release of the entire cell contents into the spur cavity contribute to the composition of the nectar that the latter contains, enriching it with cytoplasmic components. We conclude that the manner of nectar exudation may vary considerably between closely related plant species, regardless of their geographical origin and phylogeny.

## Introduction

A significant number of angiosperms present floral rewards in the form of nectar (Faegri and van der Pijl 1979), an aqueous solution consisting mainly of carbohydrates, together with other substances, such as amino acids, produced by specialized secretory tissues that together constitute the nectary (Nepi 2007). Nectaries are of great ecological importance in that they synthesize nectar that both serves to attract animal pollinators and also reinforces their behavior by functioning as a food reward (Heil 2011). For example, the position of nectaries within the flower determines the nature of plant-pollinator interactions (Pacini and Nepi 2007), and structural details are important if we are to understand how nectar is secreted and discharged or released, as well as the ecological and physiological context of nectar secretion and production.

Ranunculaceae comprises some 59 genera and approximately 2500 described species having a worldwide distribution. This family has been considered one of the most basal of eudicot families (Tamura 1993). Molecular and phylogenetic analyses strongly support the monophyly of Ranunculaceae, and this is further underscored by the irregular arrangement of stamens and the presence of more than two ovules per carpel (Wang et al. 2009). Phylogenetic analyses, however, have revealed that *Aconitum* and *Delphinium* are sister groups, the latter containing *Consolida* (Jabbour and Renner 2012b), whereas *Aquilegia* is assigned to the *I*s*opyrum* alliance (Hoot 1995).

The morphology of the petals and the entire perianth varies greatly from taxon to taxon within Ranunculaceae. Genera of this family typically have petaloid sepals, but these may vary with regard to the presence of sterile or nectariferous petals in the second whorl (Tamura 1993). The nectaries of Ranunculaceae are also very variable and defined both by differences in their number and shape (i.e., peltate, epeltate, flat, or spurred). They have also been used for delimiting subfamilies, tribes, genera, and subgenera (Bernardello 2007). In this family, nectar may be secreted either by staminodes, carpels, or the filament bases (Erbar and Leins 2013). The remaining nectariferous species, however, produce nectar in specialized nectary organs, formerly referred to as “honey leaves” or “nectary leaves” (Rasmussen et al. 2009). Although these organs are sterile and positioned in the second floral whorl and thereby correspond in position to the broader definition of petals, they are considered to have evolved independently on many occasions from stamens (Rasmussen et al. 2009; Kramer and Hodges 2010). Furthermore, in some genera, the limb of the petal increases in size and forms a visually attractive nectary spur that eventually replaces the sepal (Kosuge 1994). In Ranunculaceae, the number of nectary spurs per flower, as well as their relative dimensions, also differ significantly from species to species (Tamura 1993; Jabbour and Renner 2012a; Denisow and Antoń 2012). Thus, much of the intraspecific and interspecific variation in spur morphology found in this family is apparently determined more by pollinator-driven selection than by phylogeny (Jeppsson 2004; Kramer and Hodges 2010; Jabbour and Renner 2012a).

Various pollination syndromes are also known to occur in Ranunculaceae. For example, most species are animal-pollinated; however, a few taxa are anemophilous (wind-pollinated; Tamura 1993). Many genera (e.g., *Adonis*, *Anemone*, *Ranunculus*) have open, radiate, or actinomorphic flowers that lack obvious morphological features for pollination by specific animals, resulting in a generalist pollination syndrome (Tamura 1993). On the other hand, several genera have zygomorphic flowers (e.g., *Aconitum, Consolida*, *Delphinium*, or *Staphisagria*) and have evolved floral spurs that encourage pollinator specialization and promote diversification (Kramer and Hodges 2010). Indeed, several spurred members of Ranunculaceae (e.g., *Aconitum*, *Aquilegia*, or *Delphinium*) are almost exclusively pollinated by bumblebees (Jabbour and Renner 2012a); nevertheless, in *Aquilegia* and *Delphinium*, there is also evidence of sphingophily and ornithophily (Erbar et al. 1998). Pollination by a variety of pollinators is possible in members of Ranunculaceae because their flowers offer both nectar and copious amounts of pollen (Tamura 1993).

Despite its large number of species, studies of the floral nectaries of Ranunculaceae, and in particular anatomical investigations of the nectary spur which is such a characteristic feature of many of its members, have been neglected. Our aim, therefore, is to redress this imbalance by comparing the anatomical organization of the floral nectary spurs of several taxa (i.e., *Aconitum lycoctonum* L., *Aquilegia vulgaris* L., *Consolida regalis* Gray, *Delphinium elatum* L.) assigned to this family that differ in their flower morphology and geographical distribution.

## Materials and methods

Floral nectary spurs of four protandrous Ranunculaceae species were investigated: *A. lycoctonum* subsp. *lycoctonum* L. em*.* Koelle (*A. lycoctonum* hereafter), *A. vulgaris* L., *C. regalis* Gray, and *D. elatum* L. Nectary spurs of all species studied here were collected from the flowers in the male sexual phase, i.e., when approx. one half of anthers were dehisced. Plant material used in this study was obtained from plants growing at the Botanical Garden of Maria Curie-Skłodowska University, Lublin, SE Poland (51° 15′ 44′ N, 22° 30′ 48′ E). The position of the nectaries was determined for fresh flowers of all investigated species using an Olympus SZX12 stereoscopic microscope. The structure of nectary spurs was examined by means of light microscopy (LM), transmission electron microscopy (TEM), and scanning electron microscopy (SEM).

In each case, following macroscopic observations, floral spurs with nectaries were fixed in 2.5 % glutaraldehyde in phosphate buffer (pH 7.4; 0.1 M) for 12 h at 4 °C and washed three times in phosphate buffer. They were then post-fixed in 1 % osmium tetroxide solution for 1.5 h and washed three times in distilled water. Subsequently, the fixed material was dehydrated in a graded ethanol series and infiltrated with LR white resin (LR White acrylic resin, medium grade, Sigma-Aldrich). Following polymerization at 60 °C, ultrathin sections (60 nm) of the embedded material were cut for TEM examination using a Reichert Ultracut S ultramicrotome and a glass knife. Sections were subsequently stained with uranyl acetate and post-stained with lead citrate (Reynolds 1963). Finally, the sections were examined using an FEI Technai G2 Spirit Bio TWIN transmission electron microscope, at an accelerating voltage of 120 kV. Transmission electron microscopy images were taken using a Megaview G2 Olympus Soft Imaging Solution camera.

Semi-thin sections were also cut at a thickness of 0.7–0.9 μm using a Reichert Ultracut S ultramicrotome and a glass knife. For general histology, semi-thin sections were stained with 1 % (*w*/*v*) aqueous methylene blue-azure II solution. The presence of insoluble polysaccharides was tested using Periodic Acid-Schiff’s (PAS) reagent after blocking of free aldehyde groups. Sections were examined using a Nikon Eclipse E200 light microscope, and measurements were taken using NIS-Elements Br 2 imaging software.

The sections were also examined by means of fluorescence microscopy. In order to test for the presence of cutinized cell walls, semi-thin sections were stained with auramine O. The reaction was examined using a Nikon Eclipse 90i microscope equipped with FITC filter (EXP. 465-495, DM 505; BA 515-555). Fresh, hand-cut sections of the nectary were tested for the autofluorescence of chlorophyll within plastids by illuminating the tissue under investigation with UV light. The observations were recorded using a Nikon 90i fluorescence microscope equipped with digital camera (Nikon Fi1) and NIS-Elements Br 2 software. In each case, control sections were also used.

For SEM observations, floral nectary spurs were fixed in 2.5 % glutaraldehyde in phosphate buffer (pH 7.4; 0.1 M) at 4 °C for 12 h. The material was then washed in phosphate buffer and dehydrated in a graded acetone series. The plant material was subsequently subjected to critical point drying using liquid CO_2_, sputter-coated with gold, and examined at an accelerating voltage of 30 kV using a TESCAN/VEGA LMU scanning electron microscope.

## Results

Members of Ranunculaceae examined in this study differ both in terms of floral morphology and spur anatomy. Depending on the species, floral spurs vary in number, relative dimensions, and the location of their nectaries. Their nectary spurs also differ anatomically, especially in terms of the epidermis and cuticle, nectar-producing parenchyma, nectary vasculature, and photosynthetic parenchyma (Table 1).

**Table 1:** Characteristics of flowers and nectary spurs of four species of Ranunculaceae

Species	*Aconitum lycoctonum*	*Aquilegia vulgaris*	*Consolida regalis*	*Delphinium elatum*
Number of spurs per flower	2	5	1	2
Length of floral spurs (mm) (mean ± SD)	17.53 ± 0.56	28.48 ± 1.34	15.65 ± 1.35	25.23 ± 0.56
Dimensions of the secretory epidermis (μm)	12.31 × 12.33	7.41 × 4.88	16.08 × 11.20	18.80 × 10.17
Nectary cuticle surface	Slightly puckered	Smooth	Striate	Striate
Nectar release mechanism	Epidermal cell wall disruption	Epidermal cell wall disruption	Cuticle micro-channels	Cuticle micro-channels
Location of vascular bundles in nectary	NP	NP, GP	GP	GP
Presence of chlorophyll in nectary	−	+++	++	+
Location of starch grains in nectary	GP	NP, GP	NP, GP	NP, GP

*NP* nectary parenchyma, *GP* ground parenchyma

### *Aconitum lycoctonum*

**Fig. 1:**
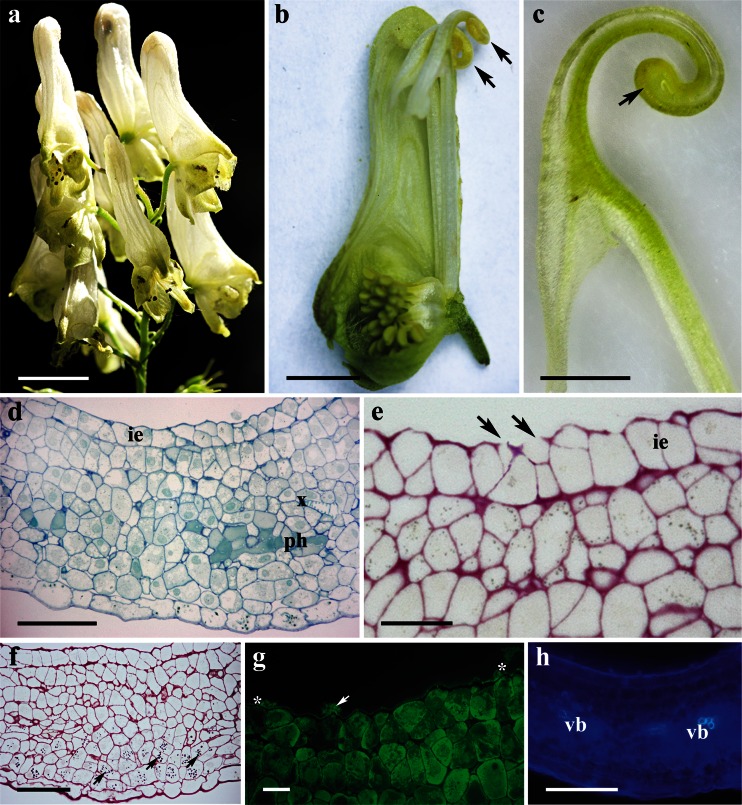
Habit of the flower and floral histology of *Aconitum lycoctonum*: **d**–**h** light micrographs. **a** Inflorescence with zygomorphic, pale yellow flowers. *Scale bar* = 10 mm. **b** Lateral view of a dissected flower with two curled nectary spurs (*arrows*) visible. *Scale bar* = 5 mm. **c** Longitudinal section through the nectary spur, note secretory area at the apex of the spur (*arrow*). *Scale bar* = 2 mm. **d** Section of nectary showing internal epidermis, underlying secretory parenchyma, and non-glandular parenchyma. *Scale bar* = 50 μm. **e** The secretory cells after PAS staining, note ruptured outer cell walls of the internal epidermis (*arrows*). *Scale bar* = 20 μm. **f** Section of nectary showing plastids with starch grains (*arrows*) and these are more abundant toward the external epidermis. *Scale bar* = 50 μm. **g** Cuticle covering internal epidermal cells that stains only slightly with auramine O, note secretory material extruding from ruptured epidermis (*arrow*), which is also positive with auramine O (*asterisk*). *Scale bar* = 20 μm. **h** Section showing lack of autofluorescence of chlorophyll in the secretory cells. *Scale bar* = 100 μm

**Fig. 2:**
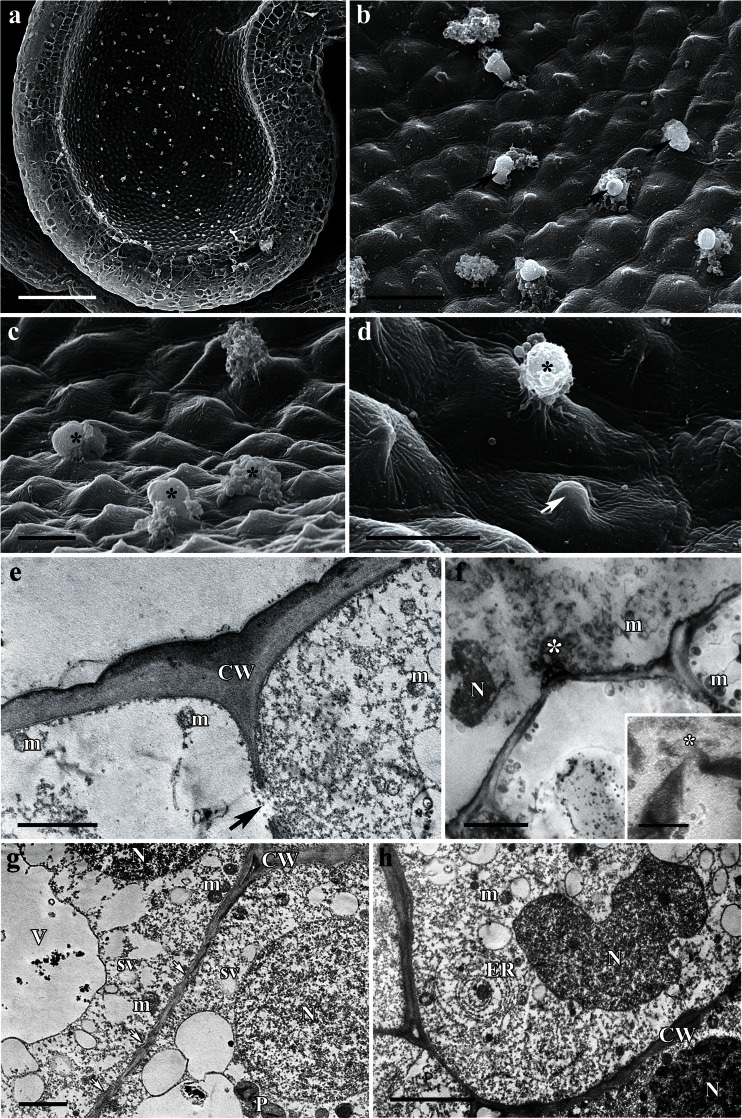
Scanning electron micrographs and ultrastructure of nectary spur of *Aconitum lycoctonum*: **a**–**d** scanning electron micrographs, **e**–**h** transmission electron micrographs. **a** Spur cut longitudinally showing spur cavity. *Scale bar* = 200 μm. **b** Glabrous surface of the internal epidermis of the nectary spur with several secreting cells (*arrows*). *Scale bar* = 20 μm. **c** Details of internal epidermis with secretory material extruded by disrupted cells (*asterisks*). *Scale bar* = 10 μm. **d** Internal epidermis with cells at different stages of secretion: Secreting cell forms blister containing cytoplasm (*arrow*), whereas another (*asterisk*) releases its secretion via the ruptured cell wall. *Scale bar* = 10 μm. **e** Internal epidermis with relatively thick outer cell wall and thin cuticle, note pit field in the radial cell wall (*arrow*). *Scale bar* = 2 μm. **f** Secretory material (*asterisk*) with nucleus, mitochondria, cytoplasm, and other disorganized organelles being visible in the spur cavity. *Scale bar* = 5 μm. The *inset* shows broken outer cell wall of the internal epidermis with a secretory material (*asterisk*) dispersing into the spur cavity. *Scale bar* = 2.5 μm. **g** Subepidermal nectariferous parenchyma cells, each containing a large nucleus, mitochondria, secretory vesicles, and plastids, note numerous plasmodesmata (*arrows*) between the radial cell walls. *Scale bar* = 2 μm. **h** Cells of the nectar-producing parenchyma with nucleus, several small vacuoles, and arrays of rough endoplasmic reticulum. *Scale bar* = 5 μm

The flowers of *A. lycoctonum* are zygomorphic, scentless, and pale yellow with a narrow corolla tube (Fig. 1a). The highly specialized perianth consists of five petaloid sepals, whereas the posterior sepal is helmet-shaped and conceals two long-stalked and curled spurs (Fig. 1b). The wall of the nectary spur is composed of several layers of cells. Nectariferous tissue is located at the apex of the spur (Fig. 1c); the surface of the nectary is glabrous, and the epidermis has a slightly puckered cuticle (Fig. 2a–d). Only the cells of the inner region of the nectary spur are involved in secretion. The nectar is apparently released via the epidermis and passes through the broken cell wall, as indicated by the flow of secreted material from the ruptured surface of the internal epidermis (Figs. 1e and 2b–d). As a result, cytoplasm, mitochondria, nuclei, and other disorganized organelles are frequently seen in the spur cavity (Fig. 2f). However, the secretory process is not simultaneous for all epidermal cells but occurs at different rates for individual cells, and consequently, it is possible to observe a range of cells, each exhibiting a different stage of nectar secretion. At first, the epidermal cells have a distended cuticle and the blister formed contains cytoplasm. As the cuticle starts to tear, the protoplasm of the underlying cell is extruded as a dense body (Fig. 2d). The secretory material stains weakly with auramine O (Fig. 1g).

The nectary consists of a single-layered internal epidermis, several layers of nectar-producing isodiametric, parenchyma cells, and underlying non-glandular parenchyma (Fig. 1d). The nectariferous cells are thin-walled and have dense cytoplasm that stains intensely with methylene blue-azure II solution. Collateral vascular bundles occur in the nectary parenchyma; phloem elements predominate (Fig. 1d). Numerous plastids with starch grains are especially abundant toward the outer epidermis (Fig. 1f). The cuticle present on the internal epidermis is thin and stains only slightly with auramine O (Fig. 1g). Autofluorescence of chlorophyll was not observed for secretory tissue and non-glandular parenchyma cells (Fig. 1h).

Ultrastructural studies revealed that secretory cells contain numerous mitochondria, plastids, rough endoplasmic reticulum (RER) cisternae, vesicles, and relatively large nuclei (Fig. 2e–h). Plastids with a dense stroma, containing osmiophilic globules, were frequently observed. Vacuoles with electron-transparent contents are small and numerous (Fig. 2g–h). The outer wall of the internal epidermal cells is relatively thick and cellulosic. The radial cell walls of the internal epidermis are relatively thin and contain numerous pit fields (Fig. 2e), but these are completely absent between adjoining parenchyma cells of the nectary. However, plasmodesmata are present, connecting cells of the nectary parenchyma (Fig. 2g), but these structures are rarely observed between parenchyma and internal epidermis of the nectary.

### *Aquilegia vulgaris*

**Fig. 3:**
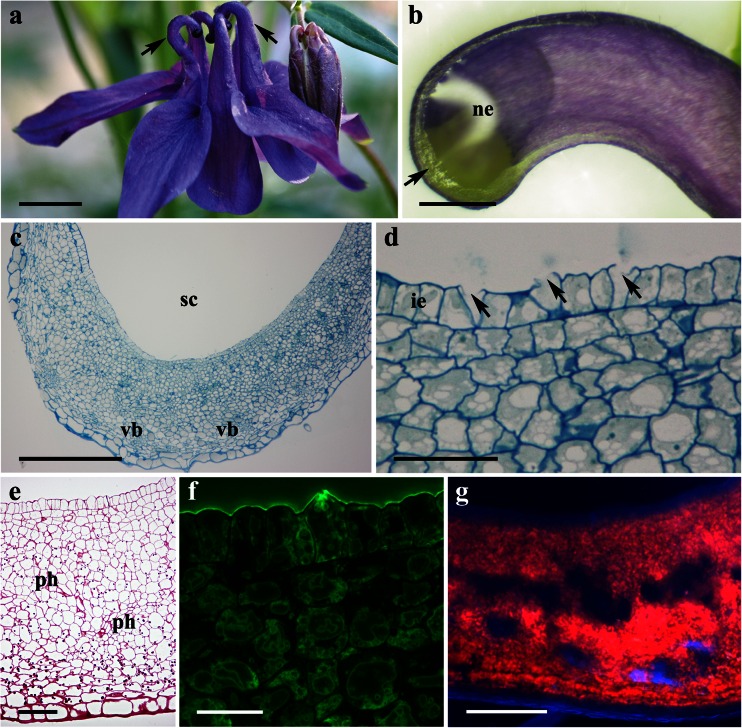
Habit of the flower and floral histology of *Aquilegia vulgaris*: **c**–**g** light micrographs. **a** Purple flower with long nectary spurs (*arrows*). *Scale bar* = 10 mm. **b** Longitudinal section through the spur showing green nectariferous tissue located at its apex (*arrow*). *Scale bar* = 1 mm. **c** Transverse section of the spur with nectariferous tissue and underlying, non-glandular parenchyma with vascular bundles. *Scale bar* = 200 μm. **d** Details of nectariferous cells with intensely staining cytoplasm and numerous vacuoles, note several internal epidermal cells with broken outer cell walls (*arrows*). *Scale bar* = 20 μm. **e** Section of nectary tissue showing nectar-producing and non-glandular parenchyma cells, both accumulating starch grains in plastids, note phloem elements penetrating nectar-producing parenchyma. *Scale bar* = 50 μm. **f** Internal secretory epidermis with a thin cuticle layer that stains only slightly with auramine O. *Scale bar* = 20 μm. **g** Strong autofluorescence of chlorophyll occurs both in nectar-producing and ground parenchyma cells. *Scale bar* = 100 μm

**Fig. 4:**
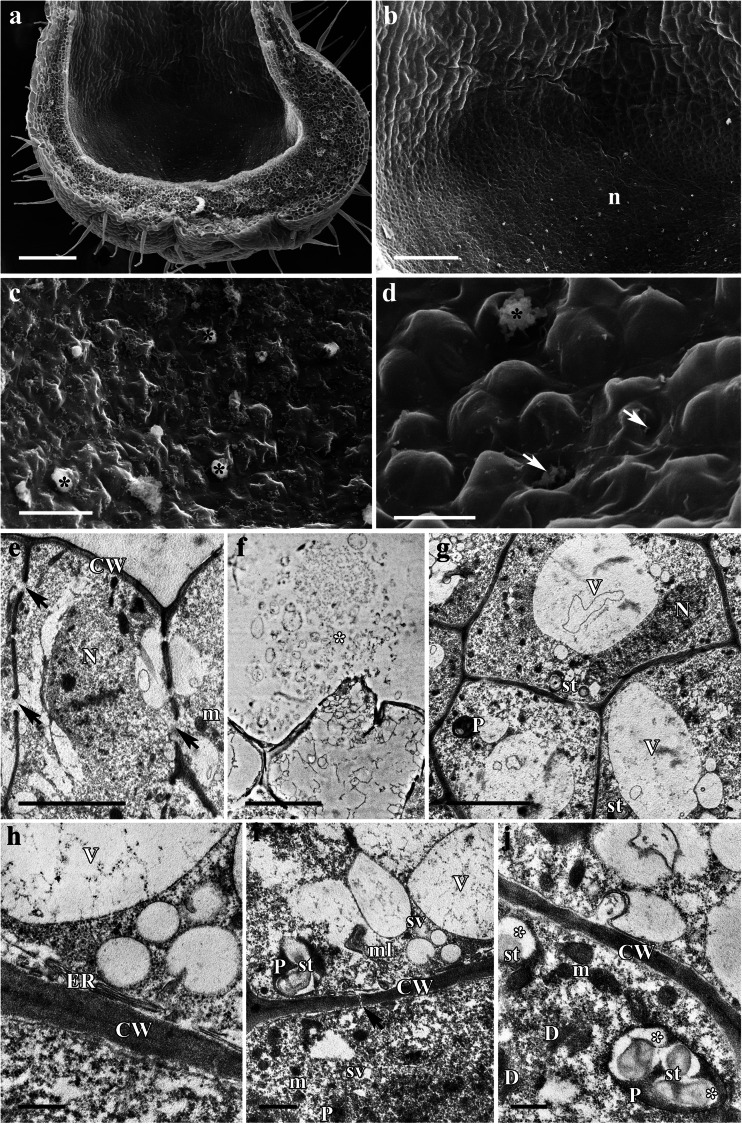
Scanning electron micrographs and ultrastructure of nectary spur of *Aquilegia vulgaris*: **a**–**d** scanning electron micrographs, **e**–**j** transmission electron micrographs. **a** Micrograph showing spur cavity. *Scale bar* = 200 μm. **b** Interface between nectariferous (n) and non-glandular surfaces. *Scale bar* = 100 μm. **c** Surface of nectariferous tissue showing several secreting cells; secreted material is present (*asterisks*). *Scale bar* = 20 μm. **d** Details of internal epidermis with extruded secretory material (*asterisk*); following the release of secretory material, depressions are evident on some epidermal cells (*arrows*). *Scale bar* = 10 μm. **e** Internal epidermal cells with large, centrally positioned nucleus and dense cytoplasm; numerous pit fields occur in radial cell walls (*arrows*). *Scale bar* = 5 μm. **f** The outer cell wall of the internal epidermis becomes ruptured, and the cytoplasm and disorganized organelles can be seen in the secreted material. *Scale bar* = 5 μm. **g** General view of nectar-secreting parenchyma showing large central vacuole and dense parietal cytoplasm with mitochondria and plastids. *Scale bar* = 5 μm. **h** Secretory parenchyma cells with parietal cytoplasm, large central vacuole, and profiles of rough endoplasmic reticulum. *Scale bar* = 500 nm. **i** Cytoplasm with mitochondria and plastids containing partly hydrolyzed starch grains, note plasmodesmata connecting adjacent cells (*arrow*) and intravacuolar myelin-like figure (ml). *Scale bar* = 1 μm. **j** Nectariferous parenchyma cells with numerous mitochondria, dictyosomes, and plastids with eroded starch grains, note the formation of electron-translucent profile within plastids (*asterisks*). *Scale bar* = 500 nm

*A. vulgaris* has actinomorphic, blue to purple, scentless flowers. The perianth consists of five petaloid sepals alternating with five petals; the latter are elongated to form showy spurred organs (Fig. 3a). The floral secretory tissue of *A. vulgaris* is developed exclusively at the apices of the inner surface of the spurs, and the light green area of the nectaries contrasts markedly with the purple non-glandular region of the spur (Fig. 3b). The surface of nectariferous tissue is glabrous, and the internal epidermis lacks stomata (Fig. 4a–d). The nectar in *A. vulgaris* is released by the rupture of individual epidermal cell walls, becoming perforated where nectar discharge has occurred. Indeed, secreted material was frequently seen to flow from the punctured internal epidermis (Fig. 4c–d), the cytoplasm and other disorganized cytoplasmic components being visible in the spur cavity (Fig. 4f). Once material is released from the epidermis, the cuticle collapses, and the secreting cells display a depression where the secreted material formerly occurred (Fig. 4d). The secretory material stains weakly with methylene blue-azure II solution (Fig. 3d).

In longitudinal section, the nectary was seen to comprise a single-layered internal epidermis, several layers (10–14) of thin-walled nectary parenchyma cells, and underlying non-glandular parenchyma (Fig. 3c). Epidermal cells, like those of the underlying nectary parenchyma, are small with dense cytoplasm and with large nuclei (Fig. 3d). Collateral vascular bundles occur in the non-glandular ground parenchyma (Fig. 3c); however, phloem elements penetrate the nectar-producing parenchyma (Fig. 3e). Plastids with starch grains occur in nectar-producing parenchyma cells, but these are more abundant in non-glandular, ground parenchyma cells. In sectioned material, the outer cell wall of the internal epidermis is covered with a thin cuticle that stains only slightly with auramine O (Fig. 3f). Both nectar-producing and ground parenchyma cells exhibit strong chlorophyll autofluorescence (Fig. 3g), but it is completely lacking for the internal epidermis.

TEM observations revealed that the cytoplasm of the secretory cells is dense and rich in ribosomes and membranous organelles (Fig. 4e–j). Profiles of RER, mitochondria, dictyosomes, plastids, and vesicles are also abundant. Plastids, having a dense stroma and containing starch grains that fill most of the organelle, occur in the nectar parenchyma (Fig. 4g, i–j). Some plastids, however, contain partly hydrolyzed starch grains. Vacuoles are numerous, often enclosing myelin-like figures (Fig. 4i). The radial cell walls of the internal epidermis have numerous pit fields (Fig. 4e). Plasmodesmata connect adjoining nectar-secreting cells (Fig. 4i), but these structures rarely occur between nectar-secreting cells and those of the epidermis.

### *Consolida regalis*

**Fig. 5:**
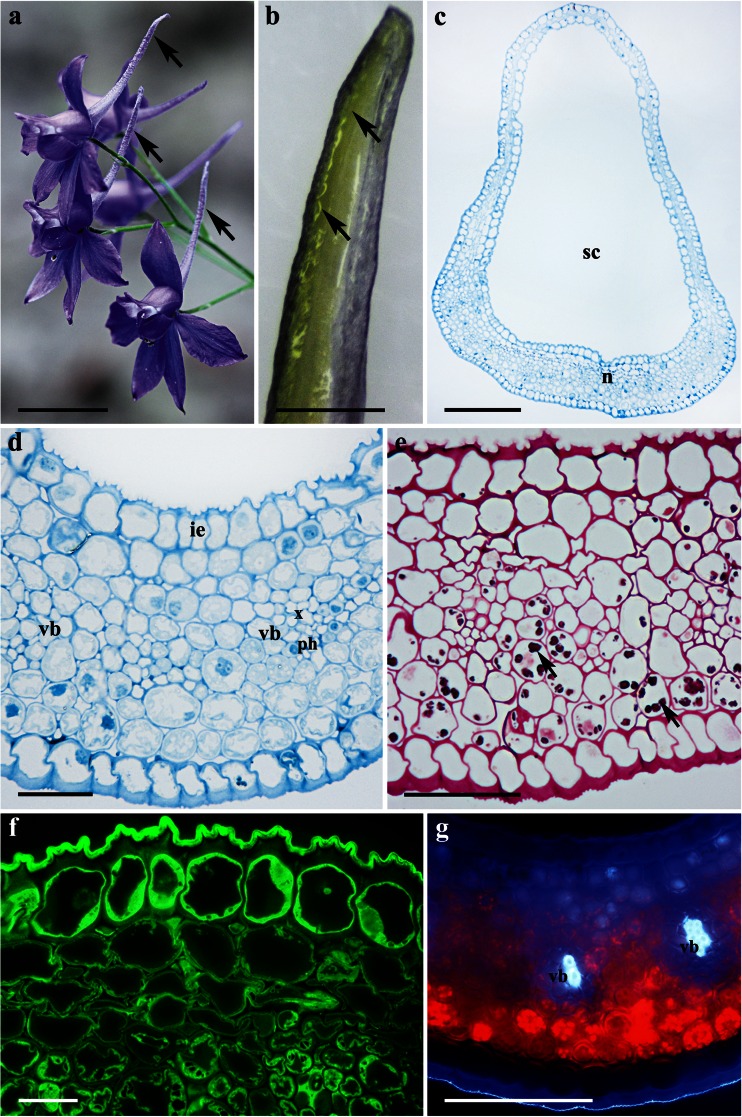
Habit of the flower and floral histology of *Consolida regalis*: **c**–**g** light micrographs. **a** Inflorescence with dark purple flowers and spurred organs (*arrows*). *Scale bar* = 5 mm. **b** Longitudinal section through floral spur showing light green nectary located at the floor of the spur (*arrows*). *Scale bar* = 0.5 mm. **c** Transverse section of the spur showing nectariferous tissue (n) and spur cavity (sc). *Scale bar* = 200 μm. **d** Section showing secretory cells with parietal cytoplasm, relatively large nuclei, and vascular bundles containing both phloem and xylem. *Scale bar* = 50 μm. **e** Parenchyma cells adjacent to sieve tubes contain numerous plastids with large starch grains (*arrows*). *Scale bar* = 50 μm. **f** Cuticle overlying internal epidermis is thick and stains intensely with auramine O. *Scale bar* = 20 μm. **g** Section showing chlorophyll autofluorescence both in secretory and ground parenchyma cells. *Scale bar* = 100 μm

**Fig. 6:**
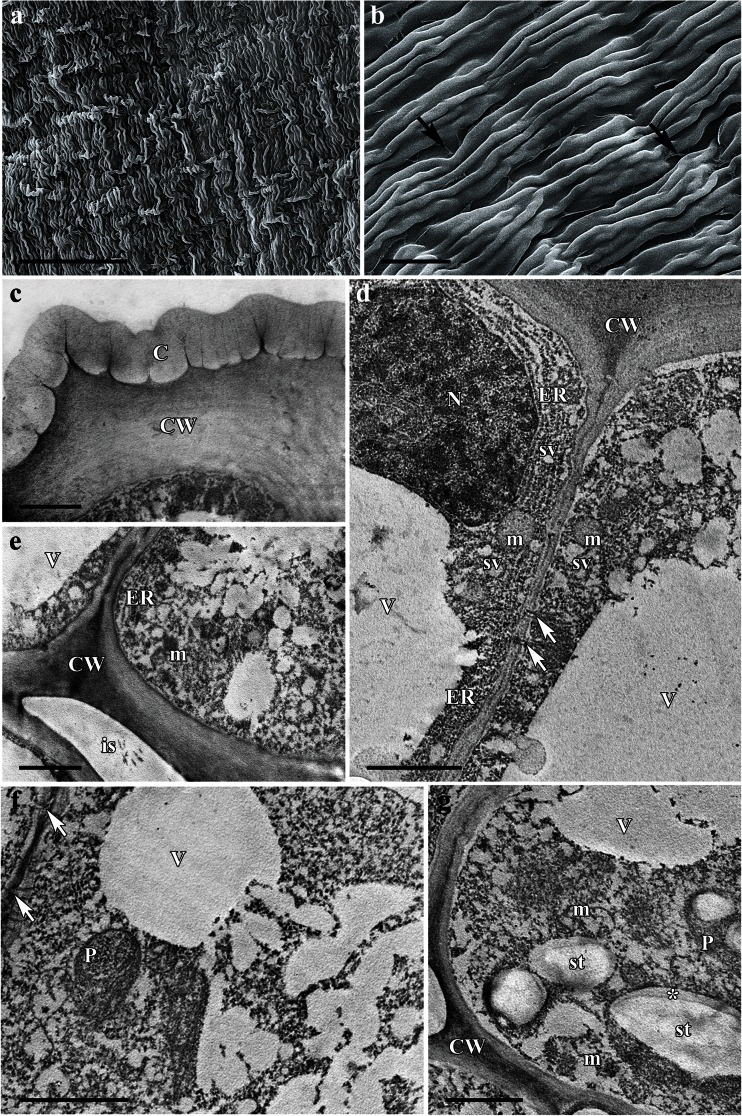
Scanning electron micrographs and ultrastructure of nectary spur of *Consolida regalis*: **a**–**b** scanning electron micrographs, **c**–**g** transmission electron micrographs. **a** Glabrous surface of inner epidermis. *Scale bar* = 100 μm. **b** Details of internal epidermis with highly striate cuticle and nectar residues (*arrows*) between rows of adjacent epidermal cells. *Scale bar* = 20 μm. **c** Details of outer tangential wall of internal epidermis with thick cuticle layer, containing numerous micro-channels. *Scale bar* = 2 μm. **d** Internal epidermal cells containing long profiles of rough endoplasmic reticulum, vesicles, large nuclei, and central vacuole, note the numerous plasmodesmata connecting the epidermal cells (*arrows*). *Scale bar* = 2 μm. **e** Section showing cytoplasm of internal epidermal cell containing mitochondria and numerous small vacuoles, note relatively large intercellular space (is) between these cells and adjoining nectary parenchyma. *Scale bar* = 2 μm. **f** Cytoplasm of secretory parenchyma cells, note numerous plasmodesmata connecting adjacent cells (*arrows*). *Scale bar* = 2 μm. **g** Secretory parenchyma with rough ER profiles and plastids containing partly hydrolyzed starch grains, note the formation of electron-translucent profile in plastids (*asterisk*). *Scale bar* = 2 μm

The flowers of *C. regalis* are zygomorphic, dark blue to purple, and lack fragrance. The double perianth is composed of five sepals and a single, spurred nectariferous petal. The latter is three-lobed and located within the spur of the dorsal, unpaired sepal (Fig. 5a). The secretory tissue is green and located along the ventral surface of the spur (Fig. 5b). The internal epidermis is glabrous and lacks secreting structures on its surface. The cuticle overlying those epidermal cells is striate and lacks evidence of rupture or pores that would allow the release of nectar (Fig. 6a–b). Nectar residues are present on the surface of the nectariferous tissue, especially in the rows of parallel adjacent epidermal cells (Fig. 6b).

The floral nectary consists of an internal epidermal layer and several (two to three) layers of nectar-producing, parenchyma cells and located beneath ground parenchyma (Fig. 5c–d). The cells of the internal epidermis, like the underlying nectar-producing parenchymatous cells, are small with dense cytoplasm and relatively large nuclei. Collateral vascular bundles occur in the ground parenchyma; phloem elements predominate (Fig. 5d–e). Parenchyma cells adjoining sieve tubes contain numerous plastids with large starch grains (Fig. 5e). The cuticle overlying the outer walls of the internal epidermis is relatively thick and stains intensely with auramine O (Fig. 5f). Autofluorescence of the chlorophyll is observed in the ground parenchyma as well as in nectariferous cells, but it is completely lacking for the internal epidermis (Fig. 5g).

Transmission electron microscopy observations revealed that cytoplasm of secretory cells is abundant with ribosomes, mitochondria, profiles of RER, and vesicles (Fig. 6d–g). The mitochondria are numerous and have an electron-translucent matrix. Dictyosomes were observed only infrequently. Plastids with partly hydrolyzed starch grains were observed in the nectary parenchyma (Fig. 6g). The cytoplasm has a well-developed vacuome, generally comprising a single large vacuole with electron-transparent content (Fig. 6d, f–g). The nectar-producing parenchyma cells have thin cell walls and relatively large intercellular spaces, whereas the outer tangential cellulose walls of the internal epidermis are thick. The cuticle overlying these epidermal cells has numerous micro-channels (Fig. 6c). Numerous plasmodesmata occur both between internal epidermal cells (Fig. 6d) and between nectar-producing cells (Fig. 6f). However, plasmodesmata also occur between the epidermis and adjoining parenchyma cells.

### *Delphinium elatum*

**Fig. 7:**
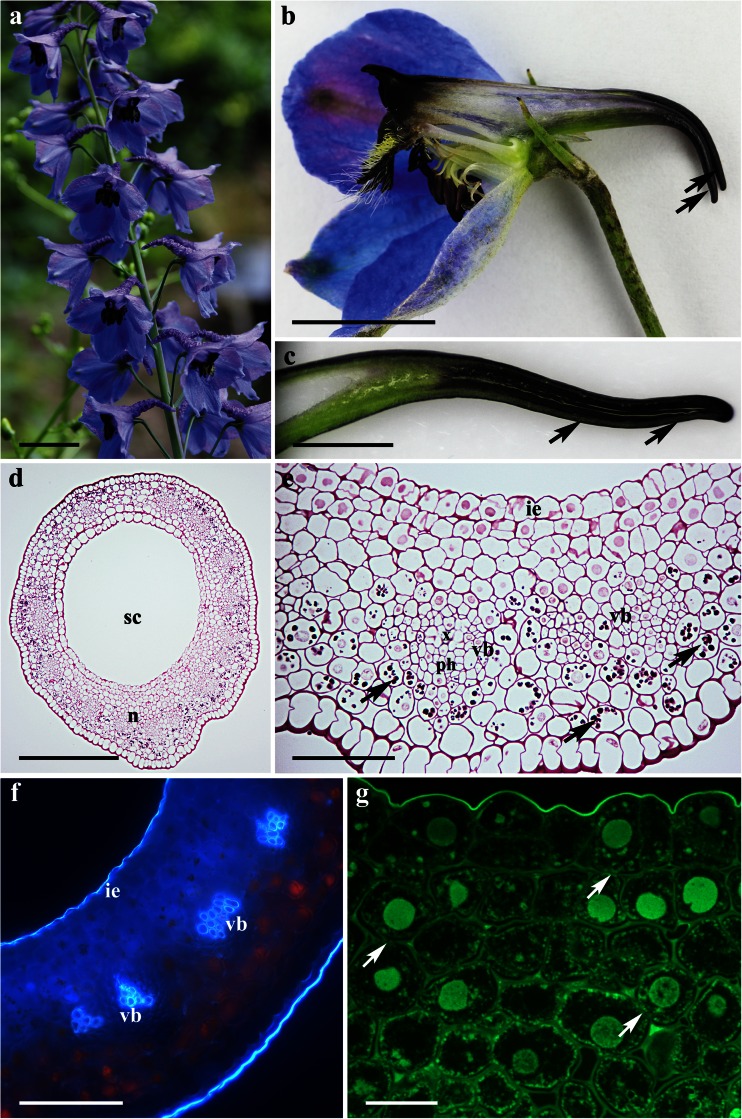
Habit of the flower and floral histology of *Delphinium elatum*: **d**–**g** light micrographs. **a** Inflorescence with dark blue flowers. *Scale bar* = 20 mm. **b** Lateral view of a dissected flower showing two nectary spurs (*arrows*) (dorsal sepal removed). *Scale bar* = 10 mm. **c** Longitudinal section through the spur showing nectariferous tissue at the ventral surface (*arrows*). *Scale bar* = 2 mm. **d** Section through the nectary spur showing spur cavity (sc) and nectariferous tissue at the spur floor (n). *Scale bar* = 200 μm. **e** Section of nectary showing internal epidermis, underlying nectariferous parenchyma cells, and vascular bundles containing phloem and xylem, located in ground parenchyma, note large starch grains (*arrows*) in parenchyma cells, especially around sieve tubes. *Scale bar* = 50 μm. **f** Red chlorophyll autofluorescence in the ground parenchyma cells. *Scale bar* = 100 μm. **g** Thick cuticle on outer walls of the internal epidermis stains intensely with auramine O, note pit fields between secretory parenchyma cells (*arrows*). *Scale bar* = 20 μm

**Fig. 8:**
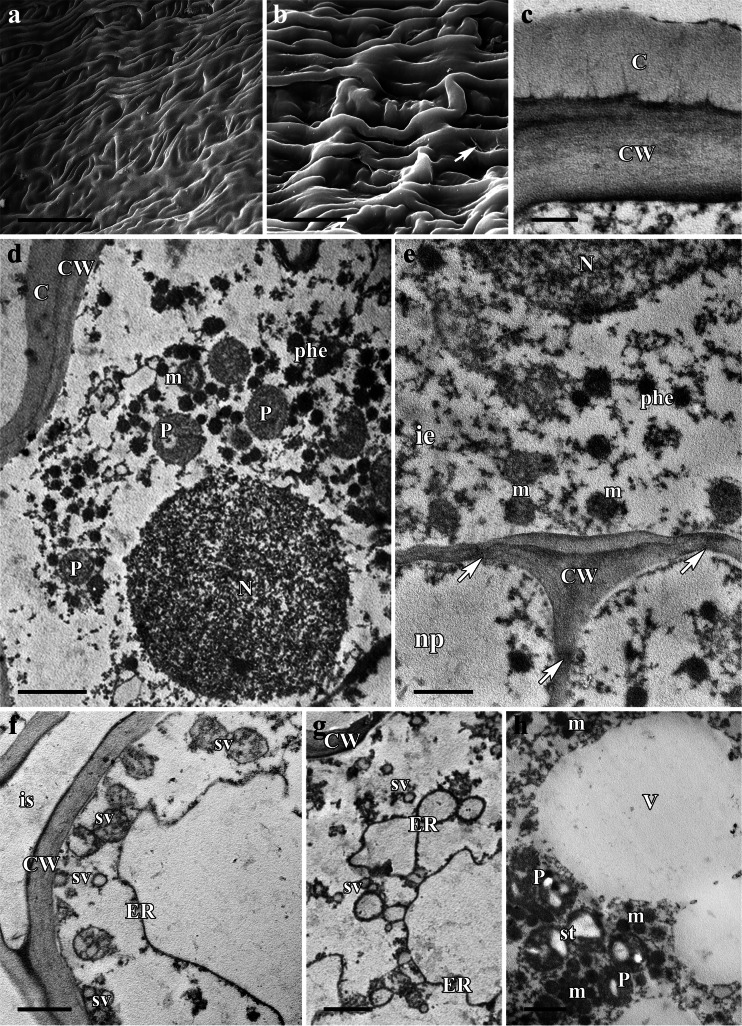
Scanning electron micrographs and ultrastructure of the nectary spur of *Delphinium elatum*: **a**, **b** scanning electron micrographs, **c**–**h** transmission electron micrographs. **a** Glabrous and striate cuticle of the inner epidermis. *Scale bar* = 50 μm. **b** Details of internal epidermis with nectary residues (*arrows*) between rows of adjacent cells. *Scale bar* = 20 μm. **c** Details of outer tangential wall of internal epidermis with cuticle containing numerous micro-channels. *Scale bar* = 500 nm. **d** Internal epidermal cell with large nucleus, mitochondria, and plastids, note phenolic comounds (phe). *Scale bar* = 2 μm. **e** Epidermal and subepidermal cells connected by plasmodesmata (*arrows*). *Scale bar* = 1 μm. **f**–**g** Nectariferous parenchyma cells containing long profiles of rough ER, note numerous secretory vesicles that coalesce and fuse with the plasmalemma. *Scale bar* = 1 μm. **h** Details of secretory parenchyma cell showing central vacuole and numerous mitochondria closely associated with starch-containing plastids. *Scale bar* = 2 μm. Abbreviations used on the figures: *C* Cuticle, *CW* Cell wall;, *ER* Endoplasmic reticulum, *ie* Internal epidermis, *is* Intercellular space, *m* Mitochondrion, *ml* Myelin-like figure, *N* Nucleus, *n* Nectary, *ne* Nectar, *np* Nectary parenchyma, *P* Plastid, *ph* Phloem elements, *phe* Phenolic compounds, *sc* Spur cavity, *st* Starch, *sv* Secretory vesicle, *V* Vacuole, *vb* Vascular bundles, *x* Xylem

*D. elatum* has zygomorphic, dark blue flowers (Fig. 7a). The perianth consists of five sepals (the dorsal sepal being differentiated to form a spur) and four petals (the two dorsal petals being elongated to form spurred organs; Fig. 7b). Longitudinal sections indicate the presence of nectariferous tissue at the floor surface of the spur (Fig. 7c). The internal epidermis lining the lumen is glabrous and its cuticle striate, lacking visible pores and cracks (Fig. 8a–b). Nectar residues occur on the surface of epidermal cells, especially in depressions between these cells (Fig. 8b).

In transverse section, the wall of the spur is composed of several layers of cells (Fig. 7d–e). The internal epidermal cells of the secretory region can be distinguished by their small size, their parietal cytoplasm, and their relatively large, centrally positioned nuclei. Beneath the internal epidermis are two to three layers of nectariferous parenchyma cells. The ground parenchyma contains several collateral vascular bundles, each containing xylem and phloem elements in equal proportions. The parenchymatous cells, especially those adjacent to the vascular bundles, contain numerous plastids with large starch grains (Fig. 7e). Ground parenchyma cells display red chlorophyll autofluorescence, but this is lacking for the nectary parenchyma and secretory epidermis (Fig. 7f). The outer, tangential walls of the internal epidermis have a relatively thick cuticle which fluoresces intensely with auramine O (Fig. 7g). This cuticular layer remains intact and has numerous micro-channels (Fig. 8c).

The cytoplasm of the secretory cells contains abundant profiles of RER, plastids, and vesicles. The cells of the internal epidermis and nectary parenchyma contain numerous mitochondria that are closely associated with plastids (Fig. 8d, h). The radial cell walls of both internal epidermis and subepidermal cells are thin, and plasmodesmata are frequent both in these walls and those of nectariferous parenchyma cells (Fig. 8e). Furthermore, relatively large intercellular spaces (Fig. 8f) occur between the cells of the layers of nectary parenchyma. Numerous secretory vesicles occur close to the plasmalemma and stages in the fusion of the vesicle membrane with the plasmalemma can often be seen (Fig. 8f–g).

## Discussion

### Nectary distribution and structure

The nectary spurs of species investigated in the present study differed markedly in length and shape, from a relatively short, single spur in *C. regalis* to two curled spurs supported on long stalks in *A. lycoctonum*, to two moderately long spurs in *D. elatum*, to five long spurs in *Aquilegia vulgaris*. According to Whittall and Hodges (2007), relationships exist between spur length and pollinator type, as floral spurs evolve and develop in a one-sided process to fit the proboscis length of the pollinator. This appears to be especially true of species examined in this study, since they all possess relatively long floral nectary spurs, and long-tongued bumblebees have already been shown to be the only visitors and/or true pollinators of all these species (Lavergne et al. 2005; Jabbour and Renner 2012a; Antoń et al. 2014). Additionally, it has been shown that pollinator selection based on spur length and/or width in the genera *Aconitum* and *Aquilegia* may differ significantly between regions and/or populations relative to the predominant guild of pollinators occurring in those regions (Jeppsson 2004; Kramer and Hodges 2010).

The floral spurs of the species studied here are directed upwards and not only serve in nectar secretion but also act as reservoirs for nectar that has already been exuded. The inner surface of the nectary spurs of all species investigated is glabrous and lacks any secretory structures. Similarly, floral nectary spurs having a glabrous inner epidermal surface have also been observed for other bee-pollinated species (e.g., Stpiczyńska et al. 2011). Moreover, whereas the cuticle of the nectary epidermis of *C. regalis* and *D. elatum* is striate, in *A. lycoctonum* and *A. vulgaris*, epidermal cells with a relatively smooth but broken cuticle were observed. Apparently, these points of rupture correspond to nectary slits observed by Erbar et al. (1998), who suggested that they play a role in nectar exudation.

The source of chemical substances used in nectar production has long been discussed (Vassilyev 2010). It is generally agreed that two alternative, non-exclusive methods of obtaining nectar carbohydrates (which, apart from water, are quantitatively the main compounds found in nectar) exist. In the first, carbohydrates are uploaded directly from phloem sap, whereas in the second, starch accumulates in plastids. The most likely theory, however, is that sugars destined to become incorporated into nectar are transported as “pre-nectar” from phloem to nectary cells either along the symplastic or apoplastic route. Although transport appears to be mainly symplastic (Nepi 2007), in the species of Ranunculaceae studied in the present paper, both pathways may co-exist, as great numbers of plasmodesmata are present in cell walls between adjoining nectariferous cells, and their relatively thin cellulosic cell walls, when tested histochemically, showed no evidence of barriers to apoplastic transport. Similar methods of sugar transport have also been proposed for the nectaries of other taxa (Heil 2011). The second method is supported by studies that reveal that nectaries may accumulate substantial amounts of starch grains in the plastids during the period directly preceding anthesis, and these are further hydrolyzed and used during nectar formation at anthesis. Alternatively, quantitative studies have shown that starch accumulation plays only a minor role in nectar production (Gaffal et al. 2007; Ren et al. 2007; Nepi et al. 2011), and this is a characteristic of floral nectaries having a short period of secretory activity (Paiva and Machado 2008). Furthermore, starchless plastids have been observed in the floral nectaries of the orchid *Ornithidium sophronitis* (Stpiczyńska et al. 2009), suggesting that phloem sap is the exclusive source of nectar sugars in that species. Indeed, certain earlier researchers (Frey-Wyssling 1955; Fahn 1979) stated that there exists a positive correlation between the amount of sugars in the nectar and the amount of phloem elements present in the nectaries. This correlation seems to be true for the species examined in this study, as the phloem elements of vascular bundles supplying the nectaries are particularly well developed. However, the role of starch accumulation in nectar formation cannot be ruled out completely for those species investigated here, as starch grains having a corroded and partly hydrolyzed surface have been observed in the plastids of nectariferous cells. It is thus reasonable to suppose that the hydrolysis of stored starch in nectary cells may yield a source of energy for highly metabolic processes and/or an additional source of nectar carbohydrates, especially when one considers the contribution of starch to the intensive production of highly concentrated nectars (ranging from 43 to 55 % of total sugars), a common feature previously recorded for the subjects of the present paper (Pyke 1978; Denisow and Antoń 2012; Antoń and Denisow 2014).

Our results also indicate that the nectariferous tissue of *A. lycoctonum* does not contain chlorophyll and is thus not capable of photosynthesis. Apparently, in that species, the complete supplies of substrates required for nectary carbon and energy metabolism and the carbohydrates secreted must be derived from distant source tissues. Indeed, a similar phenomenon, regarding transport of nectar carbohydrates from other floral or vegetative parts, has been demonstrated in a number of plant species (Pacini and Nepi 2007). On the other hand, however, in *A. vulgaris*, *C. regalis*, and *D. elatum* nectariferous and/or underlying ground parenchyma cells may directly be involved in production of nectar carbohydrates via an assimilation process, as indicated by the presence of strong chlorophyll autofluorescence on examination under UV light. Given that nectar carbohydrate production is an energy-consuming process (e.g., the cost of nectar carbohydrates can be as much as 37 % of that produced daily by photosynthesis; Pyke 1991), the participation of nectariferous tissue in nectar production is in itself advantageous, since at least a portion of nectar carbohydrates may thus originate from the photosynthetic activity of nectary cells. The presence of photosynthetic cells as components of the floral nectary has been recorded for several species (Pacini and Nepi 2007), and this phenomenon is chiefly associated with lowering the cost of flower function, i.e., reproduction and further fruit development.

The presence of dense cytoplasm, profiles of endoplasmic reticulum, and vesicles in secretory cells, as noted in the present study, is a frequently observed feature of nectar-producing tissues (Fahn 1979; Wist and Davis 2006; Stpiczyńska et al. 2011). This characteristic is also typical of granulocrine nectar formation (Nepi 2007). According to Escalante-Pérez and Heil (2012), secretory vesicles that originate from the endoplasmic reticulum and/or the dictyosomes of the nectary traverse the plasma membrane via plasmodesmata and make their way toward the cell wall.

Mitochondria often occur in groups that are closely associated with plastids. In general, these features are characteristic of highly metabolic secretory cells. As a consequence, and according to Wist and Davis (2006), these organelles interact with each other during nectar formation, suggesting exchange of metabolites and a requirement for energy as exemplified by the hydrolysis of starch grains. In addition, a greater number of mitochondria during secretory activity may also indicate that nectar formation is dependent on ATP transport, and this is frequently reflected in the mode of nectar secretion. The great number and ubiquity of mitochondria in nectariferous cells, as observed in the present paper, are similar to that demonstrated for other species (Nepi et al. 1996; Paiva and Machado 2008).

In nectariferous cells of *A. vulgaris*, vacuolar inclusions in the form of myelin-like multilamellar bodies were observed. That these inclusions originated near plasmodesmata, corroborated the findings of Eymé (1967), who suggested that they may serve as a reserve of tonoplast membranes for new vacuoles. Schnepf and Deichgräber (1984) proposed the involvement of endoplasmic reticulum in such bodies which is deposited in the vacuoles. However, Wist and Davis (2006) proposed that these inclusions may have a lysosomal function and participate in the continual degradation of senescing organelles during nectary function. Consequently, this type of inclusion has been reported both in floral (e.g., Konarska 2011) and extra-floral nectaries (e.g., Freitas and Paoli 1999) and even within vacuoles of differentiating meristematic cells of the root tip (Davies et al. 1992) where they are formed by invagination of the tonoplast and probably result from rapid membrane turnover. These observations all indicate that they have a regulatory role and should not be simply considered artifacts of tissue preparation.

### Nectar secretion and nectar release mechanisms

In the vast majority of flowering plants, nectar release occurs via modified stomata or secretory trichomes. However, the nectaries of species studied in the present paper are devoid of these secretory structures. The cuticle overlying the secretory epidermis in *A. lycoctonum* and *A. vulgaris* is relatively thin and stains only slightly with auramine O, whereas that of *C. regalis* and *D. elatum* is thick and contains much cutin and/or wax as it stains intensely with auramine O, a reagent that has a strong affinity for regions containing acidic and unsaturated waxes as well as cutin precursors (Considine and Knox 1979; Pesacreta and Hasenstein 1999). Cuticular permeability depends on the amount of wax present, and whereas a wax-rich cuticle facilitates the passage of fat-soluble compounds, it has the opposite effect on hydrophilic substances and is thus a significant barrier to the transport of water-soluble compounds (Martin and Juniper 1970). Furthermore, the cuticle of *C. regalis* and *D. elatum* lacks pores and cracks and does not become distended or detached from the outer tangential walls of the internal epidermis. Therefore, we propose that here, nectar passes along micro-channels in the cuticle covering the outer nectary surface. These micro-channels appear as fibrillar outgrowths of the outer epidermal cell wall. However, none of them appeared to have direct communication with the outside of the nectary, as reported by Koteyeva (2005) for *Helleborus foetidus*. The nectary cuticle of both species studied is striate, and it is possible that striations may facilitate nectar secretion by increasing the total secretory area. However, additional images and other techniques are required if we are to obtain a better understanding of their significance in the process of nectar exudation.

The present work indicates that the release of nectar in *A. lycoctonum* and *A. vulgaris* occurs by the rupture of the outer cell walls of internal epidermis. As a result, the contents of the secretory epidermis are discharged into the nectary spur, and thus, nectar secretion here appears to be of the holocrine type, involving death of the cell. Remarkably, there are very few reports of holocrine nectar secretion by floral nectaries, such as that demonstrated for *H. foetidus* (Vesprini et al. 2012), *Glycine max* L. (Horner et al. 2003), and *Turnera ulmifolia* L. (Elias et al. 1975). In the two latter species, however, a synchronous and massive degeneration of nectary cells occurs, and the period of nectar production lasts only ca. 24 h. Conversely, in *A. lycoctonum* and *A. vulgaris*, asynchronous, cell-by-cell degeneration of nectariferous tissue is restricted to the secretory epidermis, and the period of nectar production is much longer, lasting up to 4 and 5 days, respectively. This mode of nectar secretion is similar to that observed for *H. foetidus*, which secretes nectar over a period of about 20 days (Vesprini et al. 2012). We thus propose that in the floral nectary of *A. lycoctonum* and *A. vulgaris*, asynchronous and gradual degeneration of the internal epidermis probably provides a continuous source of nectar throughout the entire lifespan of the flower, and this is essential for the attraction of potential pollinators and effective cross-pollination.

It is also worth noting that Antoń and Denisow (2014) have recently demonstrated that significant variations in nectar carbohydrate composition between male and female sexual phases occur in the protandrous flowers of *A. lycoctonum*. The present work revealed that nectar secretion in *A. lycoctonum* is of the holocrine type. Thus, it is possible that not only protandry but also the mechanism of nectar secretion may be the cause of observable changes in the composition of nectar carbohydrate between floral sexual phases. Indeed, a similar proposal was postulated to explain intraplant variations in nectar carbohydrate composition between sexual phases of the protogynous flowers of *H. foetidus* (Canto et al. 2011). However, in the present case, a direct relationship between nectar composition and the manner of nectar secretion could not be established, and therefore, this will require further experimentation.

It is also interesting to speculate on certain aspects of the adaptation of holocrine nectar secretion to ecological processes and interactions, namely that the disruption of the cell wall and the release of the entire cell contents into the spur cavity probably enrich the nectar with cytoplasmic components. Since numerous organelles have been reported to be present in the secreted material, it is proposed that in the species studied here, transformation of cytoplasmic components occurs following disruption of the internal epidermis and the release of secretion into the spur cavity. We also propose that these additional nectar components may act as a supplementary source of nutrients not only for floral insect visitors but also for microbes present in the nectar. According to Herrera et al. (2008), foragers often carry dense aggregations of viable yeasts in their mouthparts and their nectar probing causes microbial contaminations during subsequent floral visitations. These authors also demonstrated that the metabolic activities of yeast communities may be responsible for significant changes in nectar sugar concentration and composition during the course of anthesis. Such changes to nectar composition may have a positive and/or detrimental effect on plant-pollinator mutualism and have previously been documented for other bee-pollinated Ranunculaceae species (Herrera et al. 2008; Herrera and Pozo 2010).

To conclude, our studies indicate that despite differences in floral morphology (i.e., number and relative dimensions of the nectary spurs), nectary structure of the species investigated here is moderately conservative. This similarity is expressed in the lack of secretory structures on the nectary surface, in the organization of the nectariferous tissue consisting of epidermis, underlying secretory and ground parenchyma cells, as well as in the possession of a relatively similar complement of organelles. However, there were considerable differences in the mode of nectar secretion and nectar release mechanisms. Thus, whereas in *C. regalis* and *D. elatum*, nectar was exuded through micro-channels in the cuticle, in *A. lycoctonum* and *A. vulgaris*, nectar was released by the disruption of internal epidermal cells, indicating a holocrine mode of nectar secretion. Therefore, this study shows that mechanisms involved in nectar production appear to be highly species-specific and may vary considerably even between closely related plant species. Further research is now required to improve our understanding of nectary diversity in the Ranunculaceae, its pollination ecology, and the various mechanisms used by this family to secrete and release nectar.

## References

[CR1] Antoń S, Denisow B (2014). Nectar production and carbohydrate composition across floral sexual phases: contrasting patterns in two protandrous *Aconitum* species (Delphinieae, Ranunculaceae). Flora.

[CR2] Antoń S, Denisow B, Milaniuk K (2014). Flowering, pollen production and insect visitation in two *Aconitum* species (Ranunculaceae). Acta Agrobot.

[CR3] Bernardello G, Nicolson SW, Nepi M, Pacini E (2007). A systematic survey of floral nectaries. Nectaries and nectar.

[CR4] Canto A, Herrera CM, García IM, Pérez R, Vaz M (2011). Intraplant variation in nectar traits in *Helleborus foetidus* (Ranunculaceae) as related to floral phase, environmental conditions and pollinator exposure. Flora.

[CR5] Considine JA, Knox RB (1979). Development and histochemistry of the cells, cell walls, and cuticle of the dermal system of fruit of the grape *Vitis vinifera* L. Protoplasma.

[CR6] Davies KL, Davies MS, Francis D (1992). Vacuolar development in the root meristem of *Festuca rubra* L. New Phytol.

[CR7] Denisow B, Antoń S (2012). Flowering, nectar secretion, pollen shed and insect foraging on *Aquilegia vulgaris* L. (Ranunculaceae). Acta Agrobot.

[CR8] Elias TE, Rozich WR, Newcombe L (1975). The foliar and floral nectaries of *Turnera ulmifolia* L. Am J Bot.

[CR9] Erbar C, Leins P (2013). Nectar production in the pollen flower of *Anemone nemorosa* in comparison with other Ranunculaceae and *Magnolia* (Magnoliaceae). Org Divers Evol.

[CR10] Erbar C, Kusma S, Leins P (1998). Development and interpretation of nectary organs in Ranunculaceae. Flora.

[CR11] Escalante-Pérez M, Heil M, Vivanco JM, Baluška F (2012). Nectar secretion: its ecological context and physiological regulation. Secretions and exudates in biological systems.

[CR12] Eymé J (1967). Nouvelles observations sur l’infrastructure de tissus nectarigènes floraux. Le Botaniste.

[CR13] Faegri K, van der Pijl L (1979). The principles of pollination ecology. Third revised edition.

[CR14] Fahn A (1979). Ultrastructure of nectaries in relation to nectar secretion. Am J Bot.

[CR15] Freitas L, Paoli AAS (1999). Structure and ultrastructure of the extrafloral nectaries of *Croton urucurana* Baill. (Euphorbiaceae). Bol Bot Univ São Paolo.

[CR16] Frey-Wyssling A (1955). The phloem supply to the nectaries. Acta Bot Neerl.

[CR17] Gaffal KP, Friedrichs GJ, El-Gammal S (2007). Ultrastructural evidence for a dual function of the phloem and programmed cell death in the floral nectary of *Digitalis purpurea*. Ann Bot.

[CR18] Heil M (2011). Nectar: generation, regulation and ecological functions. Trends Plant Sci.

[CR19] Herrera CM, Pozo M (2010). Nectar yeasts warm the flowers of a winter-blooming plant. Proc R Soc B Biol Sci.

[CR20] Herrera CM, García IM, Pérez R (2008). Invisible floral larcenies: microbial communities degrade floral nectar of bumblebee-pollinated plants. Ecology.

[CR21] Hoot SB (1995). Phylogeny of the Ranunculaceae based on *atpB*, *rbcL* and 18S nuclear ribosomal DNA sequence data. Plant Syst Evol (Suppl).

[CR22] Horner H, Healy R, Cervantes-Martinez T, Palmert R (2003). Floral nectary structure and development in *Glycine max* L. (Fabaceae). Int J Plant Sci.

[CR23] Jabbour F, Renner SS (2012). Spur in a spur: perianth evolution in the Delphinieae (Ranunculaceae). Int J Plant Sci.

[CR24] Jabbour F, Renner SS (2012). A phylogeny of Delphinieae (Ranunculaceae) shows that *Aconitum* is nested within *Delphinium* and that Late Miocene transitions to long life cycles in the Himalayas and southwest China coincide with bursts in diversification. Mol Phylogenet Evol.

[CR25] Jeppsson T (2004) Natural selection on floral traits in *Aconitum lycoctonum* (Ranunculaceae) in different regions of its distribution, with special regard to the presence/absence of *Bombus consobrinus* (Hymenoptera). Master thesis, Department of Studies in Biology and Environmental Sciences, Umeå University, Sweden

[CR26] Konarska A (2011). Flower nectary structure in *Cornus alba* L. Plant Syst Evol.

[CR27] Kosuge K (1994). Petal evolution in Ranunculaceae. Plant Syst Evol (Suppl).

[CR28] Koteyeva NH (2005). A novel structure type of plant cuticle. Dokl Biol Sci.

[CR29] Kramer EM, Hodges SA (2010). *Aquilegia* as a model system for the evolution and ecology of petals. Philos Trans R Soc B.

[CR30] Lavergne S, Debussche M, Thompson JD (2005). Limitations on reproductive success in endemic *Aquilegia viscosa* (Ranunculaceae) relative to its widespread congener *Aquilegia vulgaris*: the interplay of herbivory and pollination. Oecologia.

[CR31] Martin JT, Juniper BE (1970). The cuticles of plants.

[CR32] Nepi M, Nicolson SW, Nepi M, Pacini E (2007). Nectary structure and ultrastructure. Nectaries and nectar.

[CR33] Nepi M, Ciampolini F, Pacini E (1996). Development and ultrastructure of *Cucurbita pepo* nectaries of male flowers. Ann Bot.

[CR34] Nepi M, Cresti L, Guarnieri M, Pacini E (2011). Dynamics of nectar production and nectar homeostasis in male flowers of *Cucurbita pepo* L. Int J Plant Sci.

[CR35] Pacini E, Nepi M, Nicolson SW, Nepi M, Pacini E (2007). Nectar production and presentation. Nectaries and nectar.

[CR36] Paiva EAS, Machado SR (2008). The floral nectary of *Hymenaea stigonocarpa* (Fabaceae, Caesalpinioideae): structural aspects during floral development. Ann Bot.

[CR37] Pesacreta TC, Hasenstein KH (1999). The internal cuticle of *Cirsium horridulum* (Asteraceae) leaves. Am J Bot.

[CR38] Pyke GH (1978). Optimal foraging movement patterns of bumblebees between inflorescences. Theor Popul Biol.

[CR39] Pyke GH (1991). How much does floral nectar cost?. Nature.

[CR40] Rasmussen DA, Kramer EM, Zimmer EA (2009). One size fits all? Molecular evidence for a commonly inherited petal identity program in Ranunculales. Am J Bot.

[CR41] Ren G, Healy RA, Klyne AM, Horner HT, James MG, Thornburg RW (2007). Transient starch metabolism in ornamental tobacco floral nectaries regulates nectar composition and release. Plant Sci.

[CR42] Reynolds ES (1963). The use of lead citrate at high pH as an electron-opaque stain in electron microscopy. J Cell Biol.

[CR43] Schnepf E, Deichgräber G (1984). Electron microscopical studies of nectaries of some *Euphorbia* species. Akademie der Wissenschaften und der Literatur, Mainz. Trop Subtrop Pflanzenwelt.

[CR44] Stpiczyńska M, Davies KL, Gregg A (2009). Nectary structure of *Ornithidium sophronitis* Rchb. f. (Orchidaceae:Maxillariinae). Acta Agrobot.

[CR45] Stpiczyńska M, Davies KL, Kamińska M (2011). Comparative anatomy of the nectary spur in selected species of Aeridinae (Orchidaceae). Ann Bot.

[CR46] Tamura M, Kubitzky K (1993). Ranunculaceae. The families and genera of vascular plants. 2. Flowering plants. Dicotyledons. Magnoliid, Hamamelid and Caryophyllid families.

[CR47] Vassilyev AE (2010). On the mechanism of nectar secretion: revisited. Ann Bot.

[CR48] Vesprini JL, Pacini E, Nepi M (2012). Floral nectar production in *Helleborus foetidus*: an ultrastructural study. Botany.

[CR49] Wang W, Lu AM, Ren Y, Endress ME, Chen ZD (2009). Phylogeny and classification of Ranunculales, evidence from four molecular loci and morphological data. Perspect Plant Ecol Evol Syst.

[CR50] Whittall JB, Hodges SA (2007). Pollinator shifts drive increasingly long nectar spurs in columbine flowers. Nature.

[CR51] Wist TJ, Davis AR (2006). Floral nectar production and nectary anatomy and ultrastructure of *Echinacea purpurea* (Asteraceae). Ann Bot.

